# Characterization of a GDS(L)-like hydrolase from *Pleurotus sapidus* with an unusual SGNH motif

**DOI:** 10.1186/s13568-024-01752-x

**Published:** 2024-09-03

**Authors:** Miriam A. Fingerhut, Lea Henrich, Christiane Lauber, Niklas Broel, Parviz Ghezellou, Dominik Karrer, Bernhard Spengler, Kim Langfelder, Timo Stressler, Holger Zorn, Martin Gand

**Affiliations:** 1 Institute of Food Chemistry and Food Biotechnology, Justus Liebig University Giessen, Heinrich-Buff-Ring 17, 35392 Giessen, Germany; 2 Hochschule Fresenius - University of Applied Sciences, 65510 Idstein, Germany; 3 Institute of Inorganic and Analytical Chemistry, Justus Liebig University Giessen, Heinrich-Buff-Ring 17, 35392 Giessen, Germany; 4 AB Enzymes GmbH, Feldbergstrasse 78, 64293 Darmstadt, Germany; 5 Fraunhofer Institute for Molecular Biology and Applied Ecology, Ohlebergsweg 12, 35392 Giessen, Germany

**Keywords:** *Pleurotus sapidus*, GDSL, SGNH hydrolase, Heterologous expression, *Trichoderma reesei*, Enzyme characterization

## Abstract

The GDS(L)-like lipase from the Basidiomycota *Pleurotus sapidus* (PSA_Lip) was heterologously expressed using *Trichoderma reesei* with an activity of 350 U L^−1^. The isoelectric point of 5.0 was determined by isoelectric focusing. The novel PSA_Lip showed only 23.8–25.1%, 25.5%, 26.6% and 28.4% identity to the previously characterized GDSL-like enzymes phospholipase, plant lipase, acetylcholinesterase and acetylxylan esterase, from the carbohydrate esterase family 16, respectively. Therefore, the enzyme was purified from the culture supernatant and the catalytic properties and the substrate specificity of the enzyme were investigated using different assays to reveal its potential function. While no phospholipase, acetylcholinesterase and acetylxylan esterase activities were detected, studies on the hydrolysis of ferulic acid methyl ester (~ 8.3%) and feruloylated carbohydrate 5-*O*-transferuloyl-arabino-furanose (~ 0.8%) showed low conversions of these substrates. By investigating the hydrolytic activity towards *p-*nitrophenyl-(pNP)-esters with various chain-lengths, the highest activity was determined for medium chain-length pNP-octanoate at 65 °C and a pH value of 8, while almost no activity was detected for pNP-hexanoate. The enzyme is highly stable when stored at pH 10 and 4 °C for at least 7 days. Moreover, using consensus sequence analysis and homology modeling, we could demonstrate that the PSA_Lip does not contain the usual SGNH residues in the actives site, which are usually present in GDS(L)-like enzymes.

**Supplementary Information:**

The online version contains supplementary material available at 10.1186/s13568-024-01752-x.

## Introduction

The use of residual biomass for the production of daily products, food and feed is the key in the circular economy (European Commission 2021), and simultaneously partially fulfills the UN Sustainable Development Goals (El-Chichakli et al. 2016). However, the depolymerization of renewable agro-industrial residues is a challenge that can only be overcome by efficient biomass hydrolysis (Ahorsu et al. 2018). A crucial step for the leap to technological applications is the production and use of efficient enzymes, which evolution has already tailored to degrade and modify biopolymers efficiently. Especially fungi, as natural cell factories with their unique enzymes, can degrade organic lignocellulosic biomass into biogenic raw materials, which are then available as feedstocks (Filiatrault-Chastel et al. 2021). The great biodiversity of the enzymes of the phylum Basidiomycota, the “pillar” fungi, often referred to as “higher fungi”, has not yet been sufficiently characterized, and only a few Basidiomycota-derived enzymes have been commercialized thus far (Schmidt-Dannert 2016). *Pleurotus sapidus* (oyster mushroom)*,* a white-rot fungi of the phylum Basidiomycota, is an efficient lignin decomposer and able to fully degrade complex polymers (Croan 2003; Patrick et al. 2014; Ahlborn et al. 2019). Depending on the substrate, numerous enzymes are produced by this fungus to decompose lignocellulosic biomass efficiently (Zorn et al. 2005a, b). Basidiomycota produce pronounced oxidative enzymes, comprising *inter alia* laccases and peroxidases/peroxygenases as well as an extended hydrolytic system, with both of them being necessary to fully utilize renewable biomasses (Filiatrault-Chastel et al. 2021). Within the hydrolytic system, cellulases, hemicellulases, peptidases, and lipases/esterases are the main representatives of the secreted enzymes of these fungi (Filiatrault-Chastel et al. 2021). Esterases act on ester bonds (E.C.3.1) and are intensively used in various applications, such as in the synthesis of optically pure chemicals (Bornscheuer 2002) and foods (Mehta et al. 2021). In nature, a great variety of different structural folds of esterases have been reported. The best studied structural group are the enzymes with *α/β*-hydrolase fold (Ollis et al. 1992), which have a unique arrangement of *α*-helices and *β*-sheets, that is found in a variety of enzymes such as acetylcholinesterases, acyltransferases, dehalogenases, hydroxynitrile lyases, proteases, thioesterases, PETases, MHETase and others (Bauer et al. 2019). However, especially in the group of carbohydrate esterases (CEs) of the carbohydrate-active enzymes (CAZymes) (Drula et al. 2021), different hydrolase folds are present. Beside the *α/β*-hydrolase fold, right-handed *β*-helix fold, (*β/α*)-barrel fold, two-layer-sandwich fold and three-layer *α/β/α*-structure fold, also known as SGNH fold, with or without additional domains, exist (Nakamura et al. 2017). SGNH hydrolases, as a subgroup of the GDSL hydrolases, contain the amino acid motif of Ser, Gly, Asn, and His, which are not directly consecutive in the amino acid sequence (Akoh et al. 2004). Typically, Gly and Asn donate protons to the oxyanion hole with Ser at the active site (Upton and Buckley 1995). Inside the GDS(L) family, named after the amino acids motif “Gly, Asp, Ser”, often followed by Leu, in sequential order, different enzymatic activities such as thioesterase, peptidase, arylesterase, phospholipase activity, and acetylcholine esterase activity have been reported (Akoh et al. 2004). In CE family proteins, the SGNH motif can be found in CE2, CE3, CE6, CE12, CE16 and CE17 groups (Urbániková 2021). As a result of their wide range of applications, ester hydrolyzing enzymes are interesting tools for biotechnological processes and especially for food production (Raveendran et al. 2018). For example, a lipase from *Pleurotus citrinopileatus* (PCI_Lip), is used to produce special flavors in brine cheese as an alternative to pre-gastric esterases (Sowa et al. 2022). In this study, the cDNA sequence encoding for a GDS(L)-like hydrolase from *P. sapidus* (PSA_Lip) was cloned in different vectors and subsequently used for heterologous expression of the protein using *Trichoderma reesei*. To our knowledge, this is the first GDS(L)-like hydrolase from Basidiomycota produced recombinantly. Homology studies and bioinformatics analysis allow the prediction of a SGGI motif as an alternative to the SGNH in the PSA_Lip, while different enzymatic assays provided hints to unravel its potential function.

## Materials and methods

### Chemicals

Chemicals and reagents used were obtained from Carl Roth (Karlsruhe, Germany), Alfa Aesar (Karlsruhe, Germany), Applichem, (Darmstadt, Germany), Sigma Aldrich (Schnelldorf, Germany), Th. Geyer (Renningen, Germany) or Merck (Darmstadt, Germany). Chemicals and materials for electrophoresis were from Serva (Heidelberg, Germany) and Bio–Rad (Düsseldorf, Germany).

### Strains and culture methods

*Pleurotus sapidus* (DSM 8266) was obtained from the German Collection of Microorganisms and Cell Cultures (DSMZ, Braunschweig, Germany). The strain was cultivated in standard nutrition solution (SNS, 30 g L^−1^d–(+)–glucose H_2_O, 4.5 g L^−1^l-asparagine H_2_O, 1.5 g L^−1^ KH_2_PO_4_, 0.5 g L^−1^ MgSO_4_ H_2_O, 3.0 g L^−1^ yeast extract, 1 mL L^−1^ trace element solution: 5 mg L^−1^ CuSO_4_ 5 H_2_O, 80 mg L^−1^ FeCl_3_ 6 H_2_O, 90 mg L^−1^ ZnSO_4_ 7 H_2_O, 30 mg L^−1^ MnSO_4_ H_2_O, and 0.4 g L^−1^ EDTA; pH 6.0) for 8 days (24 °C, 150 rpm, 25 mm shaking diameter). For induction of lipase production, either 4% v/v Tween® 80 or 1% v/v corn oil were added to the main culture, replacing glucose as the main carbon source. *Escherichia coli* (TOP10) was obtained from Invitrogen (Karlsruhe, Germany) and was used for vector propagation. Chemically competent cells were transformed by heat shock treatment (2 min, 42 °C) according to standard protocols. Recombinant cells were cultivated in sterile LB medium (10 g L^−1^ tryptone, 5 g L^−1^ yeast extract, 10 g L^−1^ NaCl) with 150 mg L^−1^ ampicillin used as selection marker (37 °C, 225 rpm).

### Secretome analysis

Secretome analysis of *P. sapidus* was performed at Protagen Protein Services GmbH (Dortmund, Germany) by 2D electrophoresis and subsequent MALDI-MS/MS analysis. Sequence homologies of the obtained fragments were analyzed using BLAST (Altschul et al. 1990) and Pfam (Sonnhammer et al. 1997) database.

### cDNA-synthesis

To isolate RNA, the mycelium of a submersed culture containing SNS + Tween® 80 was harvested on day 5 and 100 mg was ground in liquid nitrogen with mortar and pistil. Isolation of total RNA was performed with RNeasy™ Plant Mini Kits (Qiagen, Hilden, Germany) according to the manufacturer’s instructions. The quality of the RNA was verified by an 1% (w/v) agarose electrophoresis and ethidium bromide staining. A cDNA library of *P*. *sapidus* was produced using the isolated RNA as template with the SMART™ PCR cDNA Synthesis Kit (Takara Bio Europe S.A.S., Saint-Germain-en-Laye, France) for 21 to 24 cycles (Additional file 1: Fig. S1). Amplification of a coding GDS(L)-like hydrolase sequence of *P. sapidus* from the cDNA library was performed by PCR. The primers were derived from a homologous *P*. *ostreatus* putative GDSL type lipase sequence (Genbank accession number: KAF7436219.1, Additional file 1: Table S1). Amplification of the specific cDNA was performed in an Alpha SC PCR Thermocycler (Analytik Jena, Jena, Germany). The following PCR protocol was used: 50 ng template, 5 × PCR-Puffer including dNTP’s (Qiagen), forward and reverse primer each 50 pmol, 1.25 U HotStar HiFidelity DNA-Polymerase (Qiagen), ddH_2_O ad 50 µL, 95 °C 5 min,–95 °C 1 min, 53 °C 1 min, 72 °C 90 s–for 40 cycles 72 °C 5 min. PCR products were separated electrophoretically (1.2% (w/v) agarose gel, (Additional file 1: Fig. S2), subsequently isolated from the gel using NucleoSpin Extract II Kit (Macherey–Nagel, Düren, Germany) and finally ligated (Topo TA-Cloning® Kit, Invitrogen) into the vector pCR2.1–TOPO® (Invitrogen). The plasmid DNA was replicated in *E. coli* TOP10 cells (Invitrogen), isolated from the cells and purified (NucleoSpin® Plasmid DNA Purification, Macherey–Nagel). Sequencing of the cloned cDNA was performed by Eurofins Genomics GmbH (Ebersberg, Germany). The codon-optimized PSA_Lip sequence can be found in the supplementary file.

### Enzyme production

For recombinant production of the PSA_Lip, the codon usage of the gene was adapted to the host organism *T. reesei* (a derivative of RUT C30) (Peterson and Nevalainen 2012) and an expression cassette was constructed from the adapted sequence. The expression of lipase was investigated with three different constructs. pAB500-LipPS, in which the lipase gene with its signal sequence was under control of the *cbhI* (cellobiohydolase I) promoter and the *cbhI* terminator of *T. reesei,* was used to generate strain RH32919. pAB510-LipPS, in which the lipase gene was under control of the same *cbhI* promoter and *cbhI* terminator and secretion was mediated by the *cbhI* signal sequence was used to generate strains, which are the parallel transformants from same transformation, RH32924, RH32926 and RH32927; pAB600-LipPS, in which the lipase gene without signal sequence was fused with a *cbhII* (cellobiohydrolase II) carrier was used to generate the strains RH32928 and RH32929. The fermentation process was investigated in batch and fed-batch mode. Efficient production of the lipase was achieved by optimization of the fermentation process used batch conditions (pH5.5, 28 °C, 161 h) in monosaccharide medium D5 (5% monosaccharides, 0.7% (NH_4_)_2_SO_4_, 0.3% KH_2_PO_4_) (Additional file 1: Fig. S3). The supernatants were used for determination of enzyme activity (Additional file 1: Fig. S4).

### Determination of enzyme activity

Enzyme activity was determined photometrically using a temperature controlled multi-mode plate reader (Synergy™ 2, BioTek Instruments GmbH, Bad Friedrichshall, Germany) by performing esterase and lipase activity assays with various *p*-nitrophenol (pNP) esters (Purdy and Kolattukudy 1973; Winkler and Stuckmann 1979). For the hydrolysis of pNP-acetate (pNPA), pNP-butyrate (pNPB), pNP-valerate (pNPV), pNP-hexanoate (pNPH), and pNP-octanoate (pNPO), an esterase assay was performed using the following conditions: 130 μL of potassium phosphate buffer (80 mM, pH 7.0) supplemented with 0.5% v/v Triton X-100 (without Triton X-100 for pNPA) was mixed with 20 μL of enzyme solution in a 96-well microplate, starting the reaction by adding 50 μL of substrate solution (3.5 mM of one pNP ester). The reaction was monitored for 10 min at 405 nm and 30 °C. To determine lipase activity, 50 μL of the enzyme solution was mixed with 200 μL of the substrate solution: 0.4 mM pNPP in potassium phosphate buffer (50 mM, pH 8.0), which was supplemented with 2.2 g L^−1^ sodium deoxycholate and 1.1 g L^−1^ gum arabic. The activity was measured at 37 °C for 10 min. For both assays, one unit of enzymatic activity refers to the amount of enzyme that hydrolyzes 1 μmol of pNP ester per minute under the respective conditions (esterase assay: *ε*_p-nitrophenol_ = 0.00985 L μmol^−1^ cm^−1^ (Purdy and Kolattukudy 1973), lipase assay: *ε*_p-nitrophenol_ = 0.0183 L μmol^−1^ cm^−1^ (Winkler and Stuckmann 1979)). Besides the pNP-esters, glyceryl trioctanoate (TriC8:0) (Merck, 0.12 mol L^−1^ stock solution dissolved in DMSO) as a triacylglyceride was used as substrate. The activity was measured in 5 mM HEPES buffer (pH 8.0) with a non-esterified free fatty acids (NEFA) colorimetric assay kit (Elabscience Bionovation Inc., Houston, TX, USA) according to the manufacturer’s protocol after 1, 2 and 4 h of reaction at 30 °C, 950 rpm. Phospholipase A activity was examined with the EnzChek® phospholipase A2 assay kit (Thermo Fisher Scientific, Dreieich, Germany) according to the manufacturer’s manual. PSA_Lip was also tested for choline esterase activity with an acetylcholinesterase activity assay kit (Sigma-Aldrich) following the manufacturer’s instructions. Feruloyl esterase activity was examined by two methods, a photometric assay, modified from Ralet et al. (1994) and an HPLC method as demonstrated by Linke et al. (2013). The photometric assay is based on a shift of absorption maximum from 322 nm, corresponding to methyl ferulate, to 293 nm, which indicates formation of free ferulic acid at pH 6.0. The assay mixture was composed of 50 µL enzyme solution mixed with 940 µL of 1.3 mM ferulic acid methyl ester, 1 mM sodium azide and 2% (v/v) ethanol in 100 mM MOPS buffer (pH 6.0). Heat inactivated enzyme (incubated 10 min at 95 °C) served as control. After incubation at 37 °C and 200 rpm for 72 h, a UV/Vis spectrum from 280 to 340 nm was recorded. To detect feruloyl esterase activity via HPLC–DAD (LC-20 with Degaser DG-20A5R, quaternary pump LC20AD, Autosampler SIL-20ACHT, column oven CTO-20AC, Controller CBM-20A and PDA-Detector SPD-M20A, Shimadzu, Kyoto, Japan) at 325 nm, 125 µL enzyme solution was mixed with 375 µL substrate solution (100 mM tartrate–succinate buffer pH 6.0, 3% (v/v) DMSO, 2 mM 5-*O*-transferuloyl-arabino-furanose), incubated at 37 °C for 17 h, cooled to 4 °C, and supplemented with 500 μL acetonitrile. Heat-inactivated enzyme served as a control. 1 mM sodium azide was used to inhibit laccases. Prior to analysis, the samples were filtered through 10 kDa MWCO centrifugal filter units (Merck). 10 µL of filtrate was injected into HPLC–DAD for separation of substrate and free ferulic acid using a 250/4 Nucleosil 100–5-column (Macherey–Nagel, Düren, Germany). The mobile phase consisted of 0.05% (v/v) formic acid in water (A) and 0.05% (v/v) formic acid in acetonitrile (B). The following gradient was run: 90% A (1 min), linear gradient from 90 A to 0% A over 10 min, 0% A (5 min), linear gradient from 0 to 90% A over 1 min, 90% A (2 min) at 1.5 mL min^−1^ flow (Additional file 1: Fig. S5).

The acetylxylan esterase activity of PSA_Lip (0.3 U based on pNPO activity) towards d-xylofuranose tetraacetate (4 mM) and α-d-(+)-glucose pentaacetate (4 mM, in 10% ethanol) dissolved in potassium phosphate buffer 80 mM pH 8 was determined by TLC using pre-coated plates (Silica Gel 60 F_254_, 0.25 mm; Merck) with water/methanol/ethyl acetate, 1:2:7 (v/v), as the mobile phase. Sugars were visualized with a solution containing 120 g of (NH_4_)Mo_7_O_24_ and 5 g of (NH_4_)_2_Ce(NO_3_)_6_ in 800 mL of 10% H_2_SO_4_. The fully deacetylated sugars were used as control (4 mM). A reaction with an acetylxylan esterase from *Orpinomyces* sp. (5 U, Megazyme, Bray, Ireland) was used as positive control. The reactions were carried out at 30 °C and 600 rpm (HLC Cooling-ThermoMixer MKR 23, DITABIS - Digital Biomedical Imaging Systems AG, Pforzheim, Germany) and monitored over 24 h, while samples were taken at different time points (0.25 h, 4 h, 24 h) (Additional file 1: Fig. S6).

### Protein purification and mass spectrometry-based protein identification

The *T*. *reesei* supernatant containing recombinant PSA_Lip was purified using size-exclusion chromatography (HiLoad 16/60 Superdex 200, Cytiva, Uppsala, Sweden) with 0.6 mL min^−1^ flow with potassium phosphate buffer (80 mM, pH 7.0) as mobile phase. The fractions with the highest hydrolytic activity against pNPO were collected for gel electrophoresis and subsequent mass spectrometric analyses. The fractions were concentrated via ultrafiltration using a centrifugal filter unit (10 kDa MWCO, Merck). The purified and filtered fractions were mixed with semi-native loading buffer and semi-native sodium dodecyl sulfate poly acrylamide gel electrophoresis (SDS-PAGE) (semi-native refers to: no heat denaturation, no mercaptoethanol, 50% SDS concentration in all buffers) was performed adapted from Laemmli using a 4% stacking and 12% resolving gel and Coomassie Brilliant Blue R250 staining (Additional file 1: Fig. S7) (Laemmli 1970). Respective proteins were cut from the gel and mixed with RapiGest™ SF solution (Waters GmbH, Eschborn, Germany), approx. 10 µL per 15 µg protein, and incubated for 20 min at 80 °C. After cooling, 5 µL 100 mM dithiothreitol (DTT) was added and the mixture was heated to 60 °C for 20 min. Then 5 µL of 200 mM 2-iodoacetamide (IAA) was added and the mixture was incubated for 30 min in the dark at room temperature. Afterwards, the solution was mixed with 1.5 µL trypsin (1 µg L^−1^, Promega GmbH, Mannheim, Germany) and incubated overnight at 37 °C. The samples were purified using ZipTip® filter tips as described in the instruction manual (Merck, Darmstadt, Germany). After vacuum drying, the samples were resuspended in 3% acetonitrile/0.1% formic acid (v/v in H_2_O). The resulting peptides were separated by UltiMate 3000 RSLC UHPLC system (Thermo Fisher Scientific) equipped with a Kinetix C18 column (Phenomenex, 2.6 µm, 100 Å, inner diameter 2.1 mm, 100 mm length) coupled with a Q Exactive HF-X Orbitrap (Thermo Fisher Scientific, Bremen, Germany) mass spectrometer. Chromatographic analysis was performed at a flow rate of 250 μL min^−1^ with water/0.1% formic acid (mobile phase A) and acetonitrile/0.1% formic acid (mobile phase B). The gradient elution of 60 min was performed as follows: 3% B for 5 min, followed by 3−40% B over 40 min, 40−50% B over 5 min, 50−98% B over 5 min, and re-equilibration in 3% B. The mass spectrometer was operated in data-dependent acquisition mode (top-15 DDA) with the following parameters in full MS scans: a mass range of m/z 350–1800, mass resolution of 120,000, automatic gain control (AGC) target of 3 × 10^6^, injection time (IT) of 50 ms, and MS/MS scans with mass resolution of 30,000, AGC target of 1 × 10^5^, IT of 120 ms, isolation window *m/z* 1.3, and dynamic exclusion of 60 s. The raw files were processed using Proteome Discoverer version 2.4 (Thermo Fisher Scientific) with SEQUEST and MS Amanda search engines against the PSA_Lip databases (Additional file 1: Fig. S8).

### Biochemical characterization

For detailed characterization of *P*. *sapidus* lipase (PSA_Lip), photometric esterase and lipase assays (see: Determination of enzymatic activity) were performed. For determination of temperature and pH optima, as well as stability, pNPO was used as model substrate. The optimum reaction temperature was determined by incubation of assay solutions and using the heater of the micro plate reader at the respective temperature. To evaluate the temperature stability, portions of 100 µL enzyme solution were incubated for 60 min at the respective temperature. The graphical analysis of measured activities in relation to the temperature formed the typical sigmoid curve. Its turning point can be calculated by fitting the Y-values and is equivalent to the T_50_^60^ value of PSA_Lip, which indicates a decrease of enzymatic activity by 50% after incubation at this temperature for 60 min. To determine the pH optimum, the esterase assay was performed using 100 mM Davies buffer (Davies 1959), which was set to the desired pH. PSA_Lip was incubated for 60 min at different pH values. The data obtained with different concentrations of each substrate for the Michaelis–Menten kinetics were analyzed with OriginPro® 2021 (category: enzyme kinetics, function: Michaelis–Menten, iteration algorithm: Levenberg Marquardt, without weighting). Equation for the Michaelis–Menten fit:

$$y=\frac{{v}_{max} \cdot x}{{K}_{M} + x}$$

Protein concentrations were determined by Bradford assay, ROTI® Nanoquant (by Carl Roth), following the manufacture’s manual.

### Bioinformatic and phylogenetic analysis

For better understanding of the potential classification of PSA_Lip, a PSI-BLAST (Altschul et al. 1990) search with the non-redundant protein database was conducted, where the scoring matrix BLOSUM62 and gap cost of 11 for existence and 1 of extensions with a PSI-BLAST threshold of 0.005 were used. The twelve most similar sequences to the PSA_Lip, 27 GDSL lipases from Akoh et al. (2004), and seven CE16 family proteins from http://www.cazy.org/ as well as seven *α/β*-lipases from Basdiomycota and yeast were used to perform a multiple sequence alignment with ClustalW (Thompson et al. 1994). The phylogenetic tree generated using Geneious v.9.1 (Biomatters, Ltd., Auckland, New Zealand), was inferred under the maximum likelihood (ML) (Guindon et al. 2010) by using the PhyML plugin, substation model (Le Gascuel) LG with 100 bootstraps, all other parameters were set as default (Guindon et al. 2010). The calculation of identities and similarities of the PSA_Lip was done with the EMBOSS Stretcher online tool (.ebi.ac.uk/Tools/psa/emboss_stretcher) (Myers and Miller 1988) and the consensus sequence was visualized with WebLogo v.3 (Crooks et al. 2004).

A hybrid homology model was built based on the PSA_Lip sequence using the automatic homology modelling script of YASARA v.17.1.28 (Krieger et al. 2002). This script automatically performs sequence alignments, loop building, side chain modelling, and energy minimization (Krieger et al. 2002). Herein, the script ran three PSI-BLAST (Altschul et al. 1997) iterations to build a total of 25 models based on five template structures (PDB codes: 6UQV, 6JLZ, 5XTU, 3KVN and 4HYQ). Out of the 25 models, the best parts were automatically chosen to construct a hybrid model. Structural alignments using the homology model and structures from the protein database were performed with Pymol™ v.2.3.4. The ExPASy compute pI/Mw tool (Gasteiger et al. 2005) was used to calculate molecular mass and isoelectric point of the protein. Potential homologies to carbohydrate-active enzymes were analyzed by searching the dbCAN meta server (Zhang et al. 2018) based on the PSA_Lip sequence.

## Results

### Identification of a *Pleurotus sapidus* lipase

**Fig. 1:**
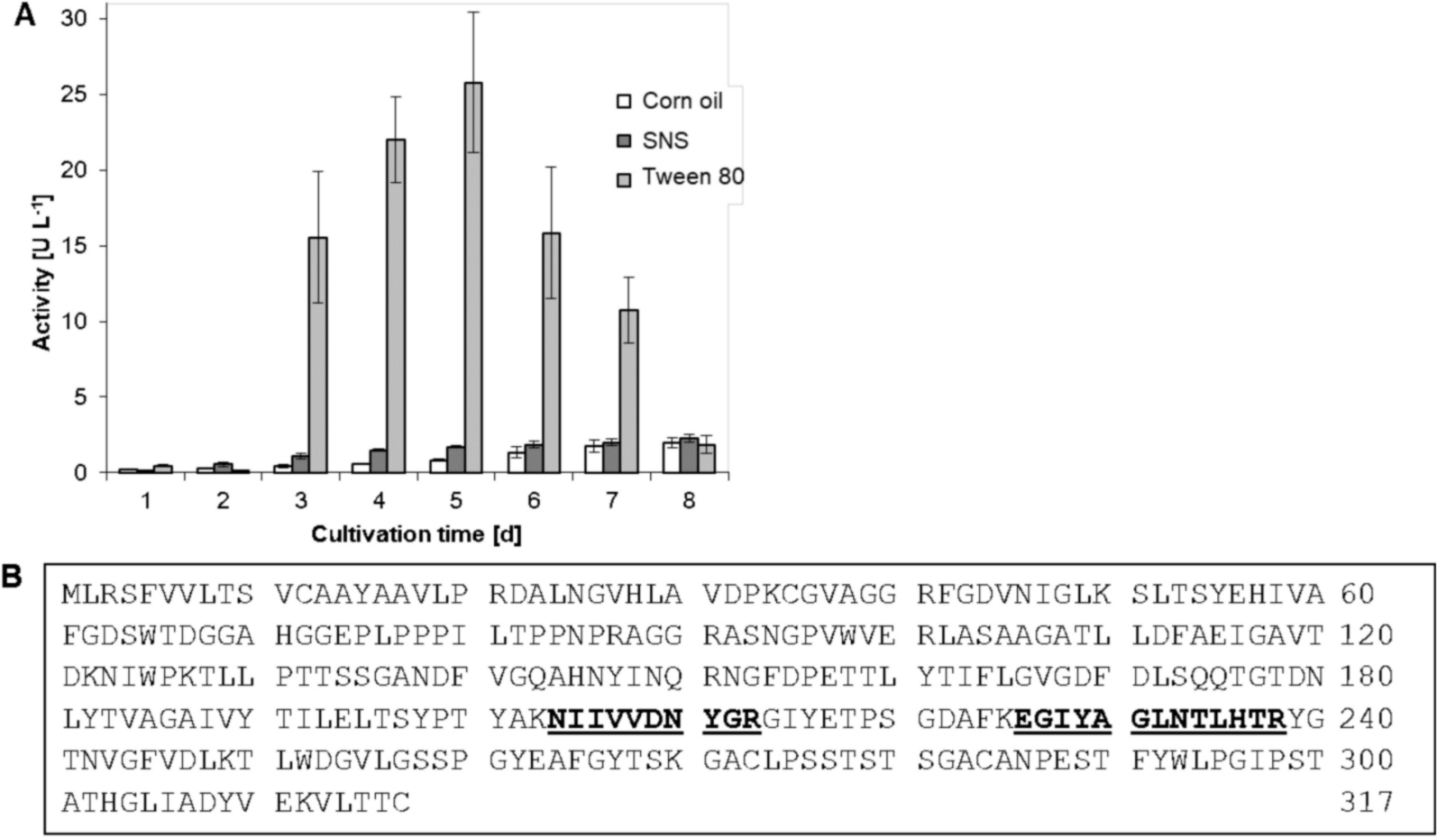
**A** Comparison of extracellular lipase activity of *Pleurotus sapidus* cultures in three different media (SNS, and SNS supplemented with either 1% (v/v) corn oil or 0.4% (v/v) Tween® 80) against pNPP on different days of cultivation. **B** Sequence of hypothetical GDSL lipase from *Pleurotus ostreatus* (Genbank accession number: KAF7436219.1). Peptide fragments identified after MALDI-MS/MS analysis of *P*. *sapidus* secretome are highlighted

In previous work, the secretome of *P. sapidus* has been mined for different enzyme activities, which for example led to the identification of a dye-decolorizing peroxidase (Lauber et al. 2017). Since there are few reports on basdiomycetous lipases (Zorn et al. 2005a, b; Bancerz and Ginalska 2007; Singh et al. 2014; Sowa et al. 2022), *P*. *sapidus* culture supernatants were also screened for respective activities using *para*-nitrophenol palmitate (pNPP) as model substrate. Monitoring of a *P*. *sapidus* culture in SNS medium over 8 days revealed a maximum activity of 2 U L^−1^ at day 8. To enhance lipase production, potential lipase substrates, either 1% v/v corn germ oil (Mazola®, Peter Kölln GmbH & Co. KgaA, Elmshorn, Germany) or 0.4% v/v Tween® 80 (Boekema et al. 2007) were added to the medium, replacing glucose as sole carbon source. While there was no significant difference with corn germ oil, Tween® 80 did indeed induce lipase formation and a maximum activity of 25.8 U L^−1^, which was observed after five days of cultivation (Fig. 1A). Consequently, a *P. sapidus* mycelium sample was taken from culture day 5 in Tween® 80 medium to isolate total RNA and to synthesize cDNA libraries (Additional file 1: Fig. S1), aiming to identify the lipase sequence. *Pleurotus sapidus* lipase specific primers for the respective gene were derived from the DNA sequence of a putative GDS(L) type lipase from *P. ostreatus* (Genbank acc. No.: KAF7436219.1) and amplified a 954 bp gene sequence (Additional file 1: Fig. S2) coding for a 317 amino acid protein, the putative *P*. *sapidus* lipase (PSA_Lip, Fig. 1B) with a calculated molecular mass of 33.5 kDa and an isoelectric point of 4.9.

### Bioinformatic analysis of PSA_Lip

**Fig. 2:**
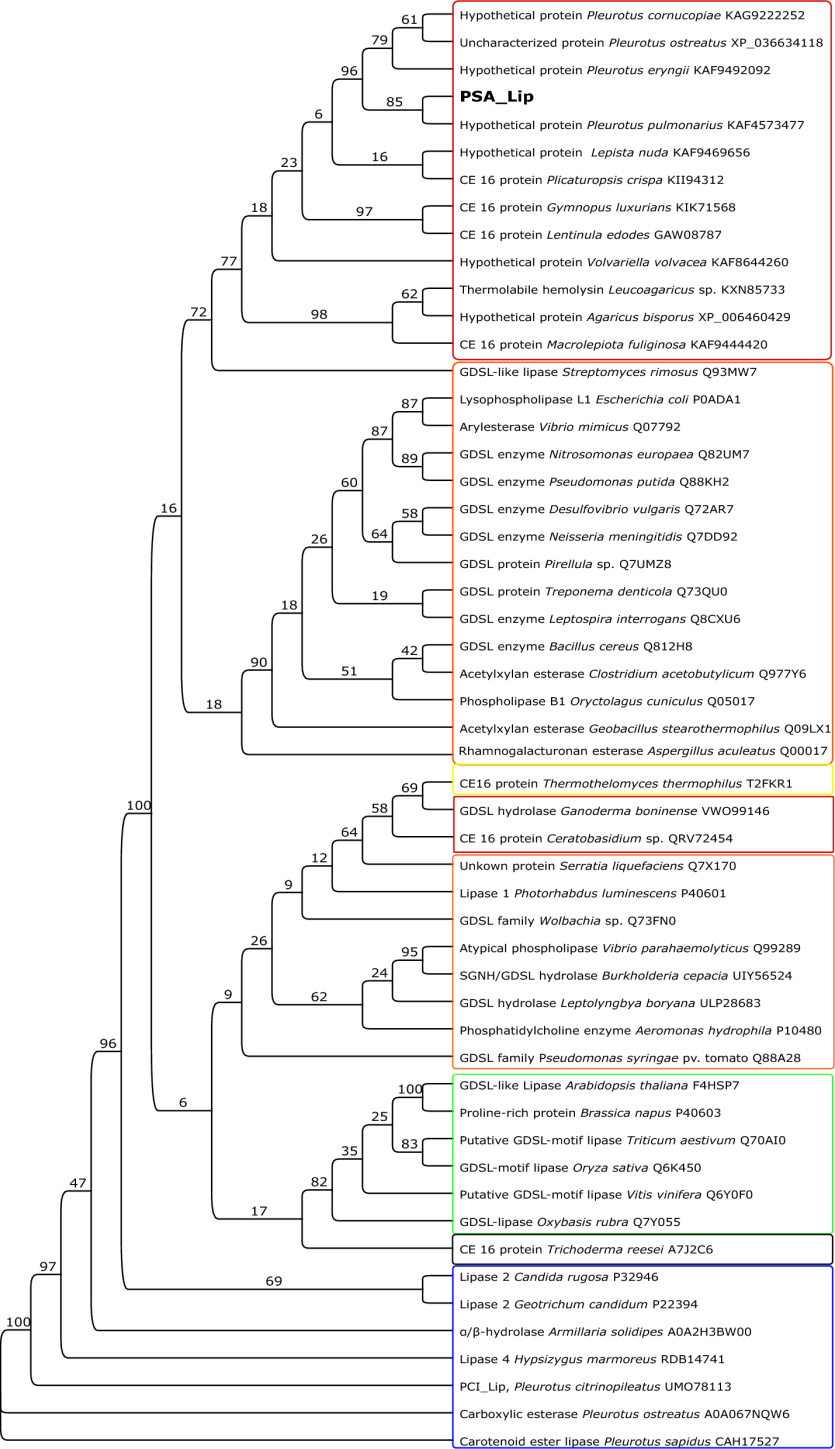
Phylogenetic protein tree based on 54 different hydrolases. The alignment was done with ClustalW. The tree was inferred under the maximum likelihood (ML) by using the PhyML plugin of Geneious v.9.1. Red frame indicates Basidiomycota origin, yellow: animal origin, black: Ascomycota origin, green: Planta origin, blue: *α/β*-Hydrolases, orange: Bacteria origin

**Fig. 3:**
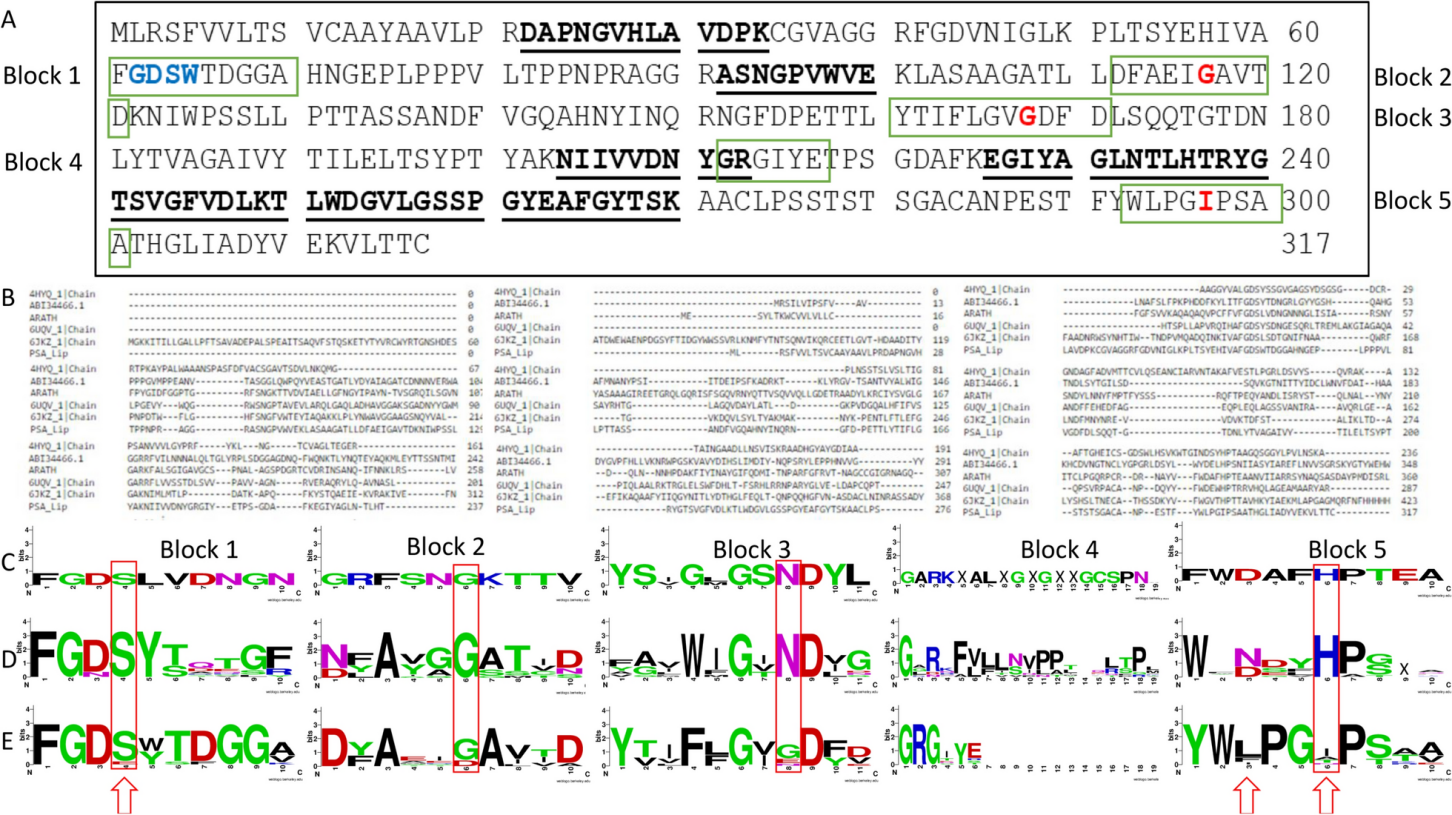
**A** Sequence of GDS(L) lipase PSA_Lip (Genbank accession number: CFW94144.1). Peptide fragments identified after LC-ESI-HR-MS/MS analysis of purified *Trichoderma reesei* supernatants are highlighted in bold and underlining. Blue font indicates the GDS(L)W motif, whilst S64 also is part of the SGGI motif (corresponding to SGNH in other hydrolases), which is highlighted in red, green blocks indicate the five consensus blocks from plant GDSL lipase. **B** Sequence alignment with ClustalW of the PSA_Lip with a GDSL lipase from *Arabidopsis thaliana*, two bacterial phospholipases (PDBs: 6JKZ and 4HYQ), one acetylcholinesterase (PDB: 6UQV) and one CE16 acetylxylanesterase of *T. reesei*, demonstrate the large differences. **C** Consensus analysis of the five blocks from plant GDSL lipases, **D** consensus analysis of the five blocks from CE16 GDS(L) hydrolyses from the cazy.org, **E** consensus analysis of the five blocks of PSA_Lip-like GDS(L) hydrolyses, Red squares indicated the SGNH motif, and the red arrows the catalytic S, D and H triade from the usual GDSL hydrolyses, x = no consensus amino acid at this position, no amino acids (in Block 4 of E) = part is missing in the PSA_Lip-like hydrolyses

For identifying similar enzymes, a PSI-BLAST (Altschul et al. 1997) similarity search was performed, which identified only experimentally uncharacterized enzymes from different Basidiomycota. However, some of them were predicted to fall into the CE 16 family of enzymes with acetylesterase activity, based on the BLAST entries. Encouraged by that, the dbCAN meta server (Zhang et al. 2018) was used with the PSA_Lip sequence as query yielding no result for any potential CAZymes, neither with the HMMER search nor with the DIAMOND algorithm. Then, seven different CE 16 family protein sequences and as well as 27 different GDSL lipases (Akoh et al. 2004), and, as outliners known *α/β*-lipases from Basdiomycota and yeast, were downloaded to analyze their phylogenetic relations (Fig. 2). As expected, the most similar proteins to the PSA_Lip group clearly clustered together (upper red box in Fig. 2), while the GDSL lipase from Akoh et al. used in this study clearly group in three groups, which can be defined as plant-based group (green box in Fig. 2) and two bacterial groups (orange boxes in Fig. 2). The CE 16 family proteins from the CAZyme database are not grouping together. This indicates that the PSA_Lip and similar enzymes are different from the currently classified GDSL enzymes and especially from the known CE16 family proteins. The consensus sequence block analysis yielded clear differences in the sequences (Fig. 3).

**Fig. 4:**
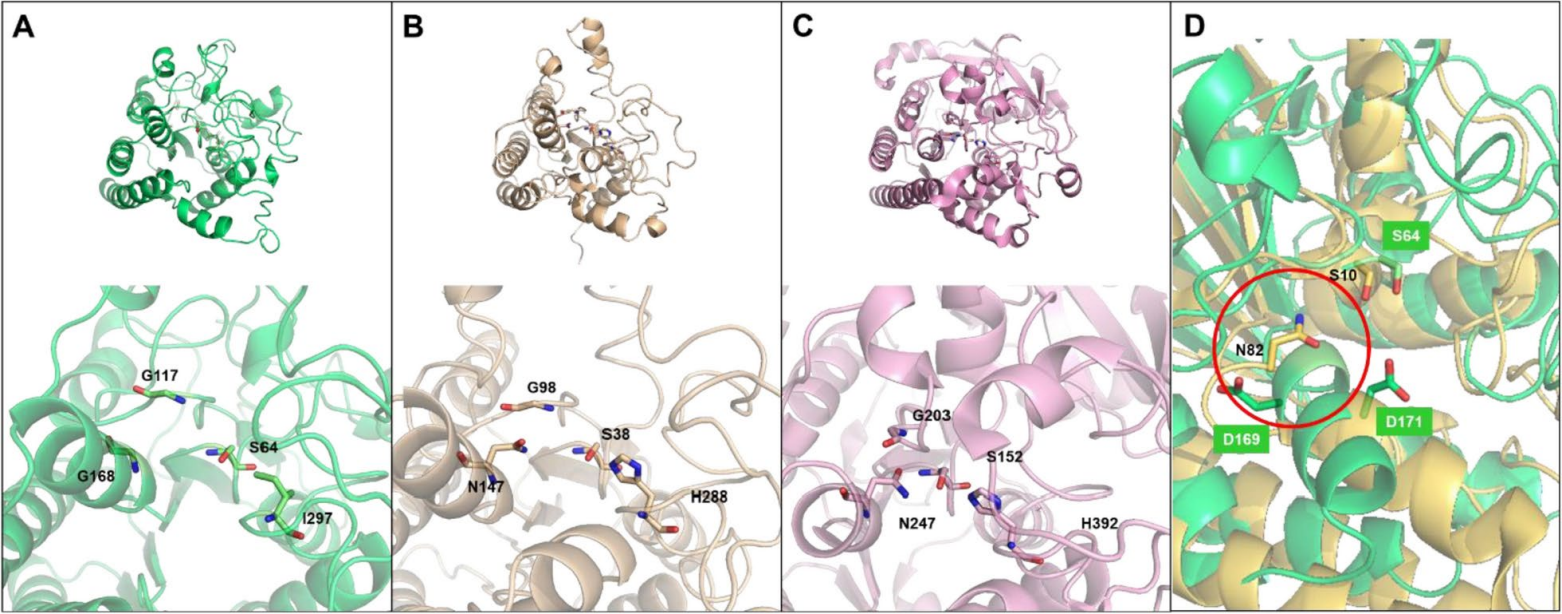
**A** Homology model of PSA_Lip and enlarged detail of the putative active site with labeled SGGI-residues. **B** Structure of a bacterial acetylcholinesterase from *Pseudomonas aeruginosa* (PDB: 6UQV) and enlarged detail of the active site with labeled SGNH-residues. **C** Structure of a bacterial phospholipase A2 from *Vibrio vulnificus* (PDB: 6JKZ) and enlarged detail of the active site with labeled SGNH-residues. **D** Structural alignment of the extracellular lipase from *Streptomyces rimosus* (PDB: 4HYQ) in yellow with the PSA_Lip in green, residues marked in white for the PSA_Lip or black for the 4HYQ, the red circle marks the shorted helix in the 4HYQ compared to the homology model of the PSA_Lip

To gain more insight into its structure-to-function relationship, 3D homology models of PSA_Lip were built. The closest PSI-BLAST hit and thus the underlying template structures were a choline esterase from *Pseudomonas aeruginosa* with PDB code 6UQV, and a phospholipase A from *Vibrio vulnificus* (PDB code: 6JKZ), though sequence homologies (Myers and Miller 1988) to PSA_Lip of 36–40% were in a medium range. Sequence identities were determined to be 26.6% to 6UQV and 23.8% to 6JKZ using EMBOSS Stretcher online tool. Therefore, the quality of the resulting model was relatively poor, with a Z-score of − 2.120. All of the template structures are also part of the SGNH hydrolase family and hence contain a GDS(L)-like motif, so the structures and, specifically, active sites were compared to the homology model. As shown in Fig. 4, the overall structure of PSA_Lip (Fig. 4A) is more similar to a *P*. *aeruginosa* cholinesterase (Fig. 4B) than to the *V*. *vulnificus* phospholipase, (Fig. 4C), which possesses a *β*-sheet fold in the upper right part that the other two structures are missing. The active site region, which is located on the lower left part of the overall structures and framed by *α*-helices, seems to have a similar geometry for all three enzymes. When focusing on the active side residues, the typical SGNH motif, consisting of S38-G98-N147-H288 and S152-G203-N247-H392 in 6UQV and 6JKZ respectively, is obvious (Fig. 4B, C). In contrast, the same motif cannot be found in PSA_Lip. The putative catalytic nucleophile S64 and G117 are roughly in the same position, but no asparagine and histidine or residues, which can fulfil the same role, could be found within a close distance to those residues in the generated model. When comparing the residue positions, PSA_Lip appears to rather have a SGGI motif, consisting of S64-G117-G118-I297, then the expected SGNH motif.

### Heterologous expression and purification

**Fig. 5:**
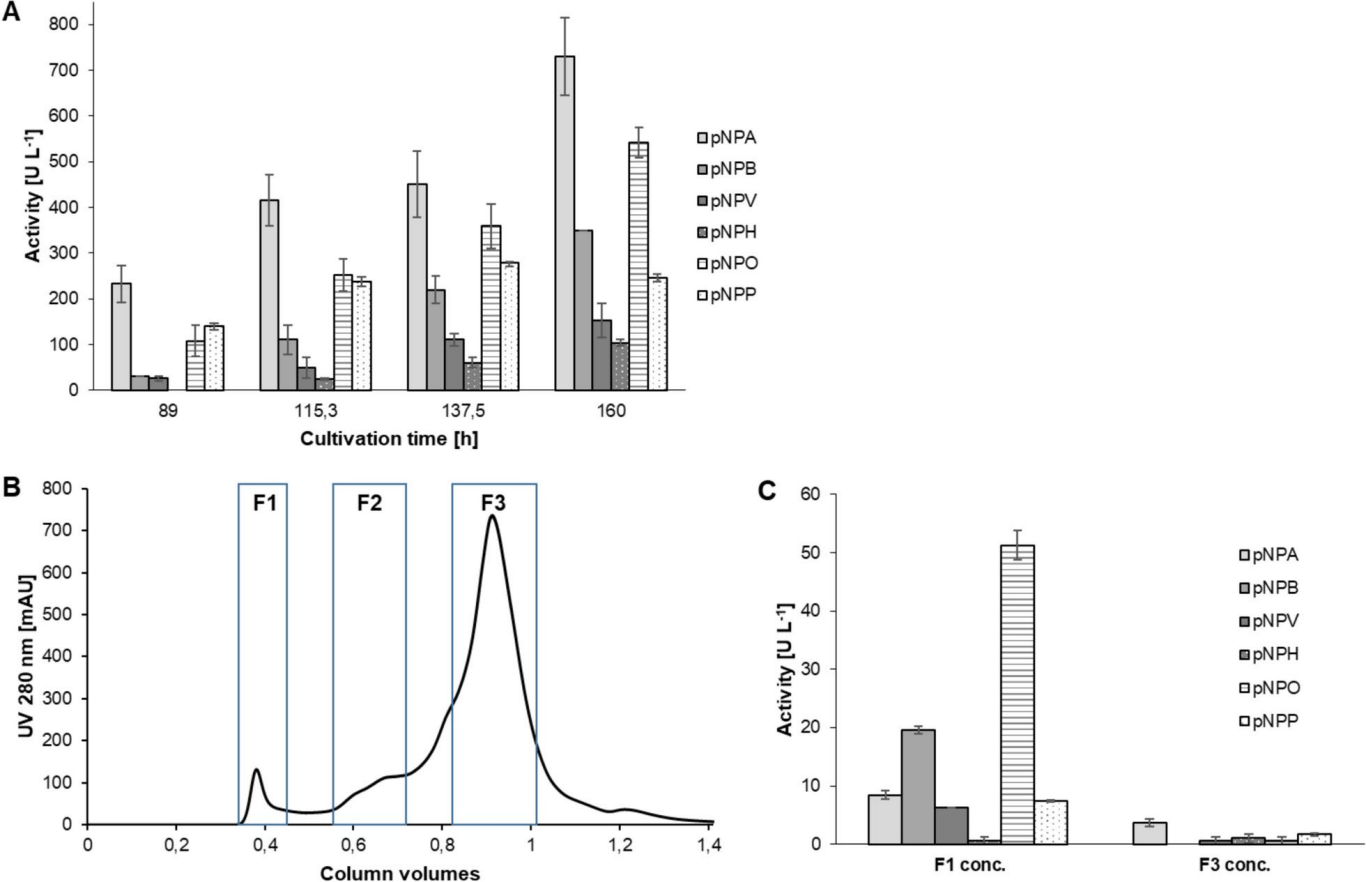
**A** Hydrolysis profiles of *T*. *reesei* culture supernatants samples during PSA_Lip expression at different time points of the cultivation. **B** Size exclusion chromatography for purification of PSA_Lip using HiLoad 16/60 Superdex 200 column. For the framed sections the fractions were pooled to samples F1, F2 and F3 for further analyses. **C** Hydrolysis profile of SEC purified samples F1 and F3 after ultrafiltration and concentration (10 kDa MWCO). F2 did not display significant hydrolytic activities

The sequence coding for PSA_Lip was transferred to AB Enzymes GmbH (Darmstadt, Germany) to establish heterologous expression in *T. reesei*. In total, six lipase-positive strains were cultivated in 0.5-L bioreactors (Additional file 1: Fig. S3). The strain RH32919, containing the construct pAB500-LipPS, reached maximum lipase activity of 360 U L^−1^ against pNPP after 161 h in medium D5. With RH32939 (construct pAB510) and RH32924 (pAB600-LipPS), maximum activity was observed in medium D5 after 137 h with 340 U L^−1^ or 280 U L^−1^, respectively (Additional file 1: Fig. S4). During a second expression in strain RH32919, samples of the supernatant were taken after 88.2 h, 115.5 h, 137 h and 161 h. The hydrolysis profile towards different pNP-esters was determined for each sample (Fig. 5A). In all samples, the maximum activities were reached after 161 h, except for lipase activity against pNPP, which slightly decreased after 137 h. Overall, the activities towards pNPA (729 U L^−1^), pNPB (350 U L^−1^) and pNPO (540 U L^−1^) were the highest at 161 h. The same samples were also used to examine feruloyl esterase activity of PSA_Lip, but only very low conversions were detected via photometric assays and HPLC–DAD (Fig. S5). Hence, these samples were used to purify PSA_Lip from *T*. *reesei* supernatant to further investigate the enzyme. Size-exclusion chromatography was performed, and the eluted fractions were then merged into three major fractions (F1, F2 and F3) based on the UV chromatogram (5B). After ultrafiltration, hydrolysis profiles were determined for the three samples (Fig. 5C). Whereas F2 did not display significant esterase activity, F1 and F3 displayed distinct differences. Whilst F1 showed highest activities against pNPB (19.6 U L^−1^) and pNPO (51.3 U L^−1^), the best substrate for F3 was pNPA (3.7 U L^−1^), indicating that PSA_Lip is contained in F1. The purification factor of F1 was determined to be 36.7 (Table 1).

**Table 1:** Specific activities towards pNPO and purification factors for PSA_Lip

Sample	Specific activity [U mg^−1^]	Purification factor
*T*. *reesei* supernatant (161 h)	0.076	1
SEC F1	2.797	36.7
SEC F3	0.059	0.6

A subsequent semi-native SDS-PAGE (Additional file 1: Fig. S7B) did not show a protein band at the expected size of ca. 33.5 kDa in F1. Therefore, the two bands in F1 (F1_1 with ~ 118 kDa and F1_2 with > 212 kDa) and one band in F3 (at the expected size) were cut, and after tryptic digestion subjected to liquid-chromatography high-resolution tandem mass spectrometry (LC-HR-MS/MS) analysis to confirm the presence of PSA_Lip. As shown in Figs. S7 and S8, the five peptide fragments of the F1_1 band showed PSA-Lip sequence similarity with a sequence coverage of 24.6% (Fig. 3A, Fig S7–8).

### Biochemical characterization

**Fig. 6:**
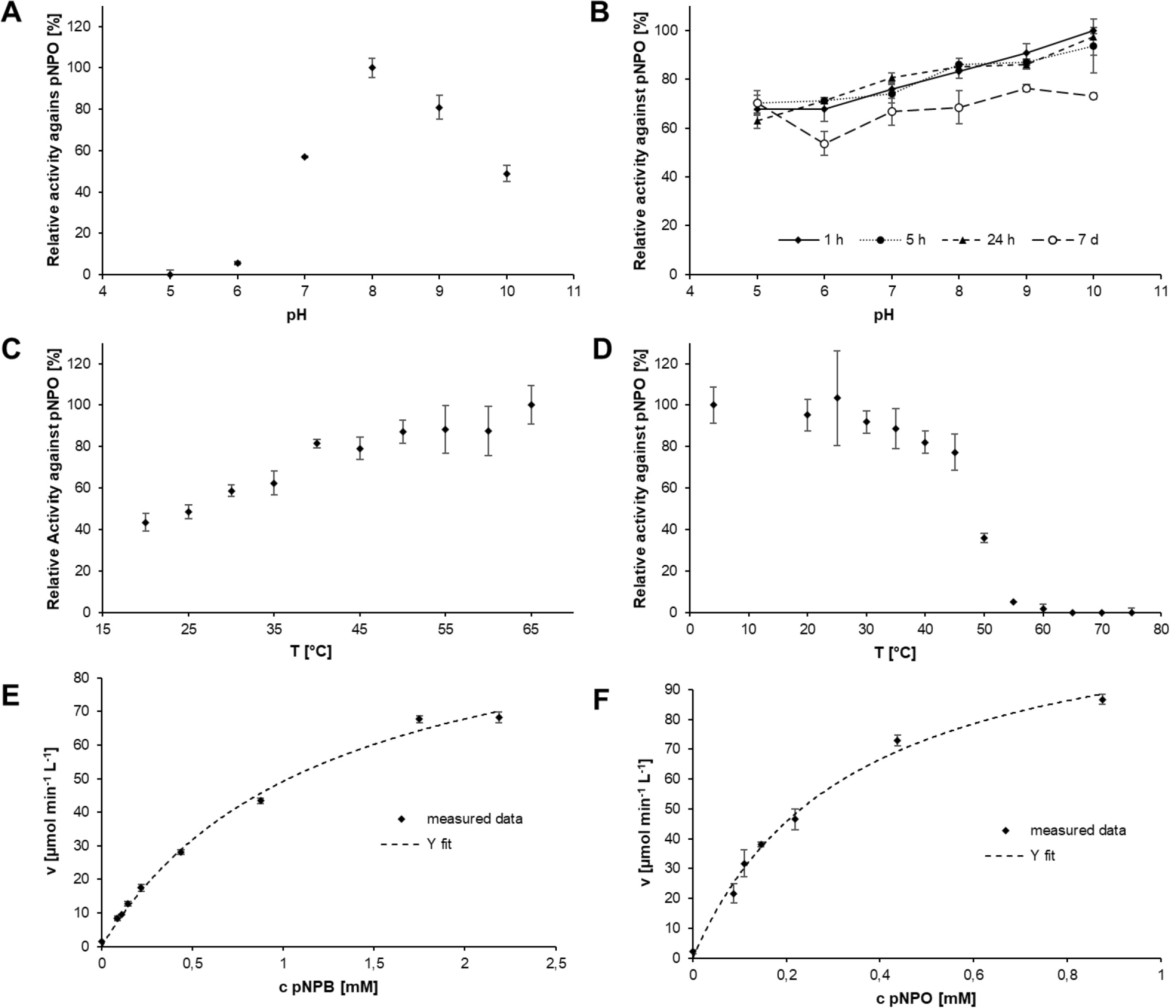
**A** Optimum pH of PSA_Lip. **B** pH stability of PSA_Lip after 1 h, 5 h, 24 h and 7 d of incubation at the respective pH and 4 °C. **C** Effect of temperature on PSA_Lip activity. **D** Temperature stability of PSA_Lip after incubation of 1 h at the respective temperatures. All four characteristics have been determined by measuring activity towards pNPO. **E** Saturation curves of PSA_Lip toward pNPB and **F** pNPO. Activity data were fitted via OriginPro® 2021 based on the Michaelis–Menten kinetics model and equation

Samples of PSA_Lip culture supernatant were also used for electrophoretic analyses to explore more preliminary characteristics of PSA_Lip, by isoelectric focusing, followed by activity staining with *α*-naphthyl acetate, again combined with Coomassie staining, an estimated pI of 5.0 was determined (Additional file 1: Fig. S9), which also supported the in silico result of pI 4.9. To characterize the purified PSA_Lip, the effects of pH and temperature were investigated using pNPO as a model substrate, as the activity towards this substrate was the highest. The optimum pH value was determined to be pH 8 (Fig. 6A). Storing the enzyme at 4 °C in buffer with different pH values revealed high pH stability. When stored at a range from pH 5 to pH 10, PSA_Lip retained at least 53% of activity, even after seven days of storage. At pH 10, the enzyme was most stable for at least 24 h and, after seven days, still 73% of the initial activity was detected (Fig. 6B). The optimum reaction temperature was at 65 °C (Fig. 6C) and the T_50_^60^ value of PSA_Lip was determined to be 48.5 °C. After 60 min of incubation at 65 °C, no residual activity could be detected (Fig. 6D). Kinetic parameters of PSA_Lip were examined for the model substrates pNPB, pNPO and pNPP. With the substrates pNPB and pNPO, Michaelis–Menten kinetics were observed by outlining saturation curves for the substrates (Table 2). The catalytic efficiency (k_cat_ K_M_^−1^) towards pNPO with 6556 s^−1^ mol^−1^ L was roughly a quadruple of the respective parameters for pNPB (1607 s^−1^ mol^−1^ L), which corresponds well to the hydrolysis profile (Fig. 6E, F). Interestingly, it was not possible to fit the data for pNPP according to the Michaelis–Menten equation, so no kinetic data could be calculated (Additional file 1: Fig. S10). With regards to the bioinformatic analyses, it was examined whether the enzyme has (acetyl-)cholinesterase or phospholipase A2 activity, though no significant activity was detected with the respective assays. Moreover, no acetylxylose or acetylglucose esterase activity for the PSA_Lip was found (Additional file 1: Fig. S9), while low activity for ferulic acid methyl ester (~ 8.3% conversion) and the feruloylated carbohydrate 5-*O*-transferuloyl-arabino-furanose (~ 0.8% conversion) was found. The PSA_Lip released octanoate for the entire investigated time, while the highest activity of 11.2 ± 1.6 mU mg^−1^ was present after 1 h (Table S2).

**Table 2:** Apparent kinetic constants of the purified recombinant lipase from *P. sapidus*

Substrate	v_max_ [µmol min^−1^ L^−1^]	K_M_ [mmol L^−1^]	k_cat_ [s^−1^]	k_cat_ K_M_^−1^ [s^−1^ mol^−1^ L]
pNPB	108.98	1.22	1.95	1607
pNPO	122.27	0.33	2.19	6556

## Discussion

The identified PSA_Lip hydrolytic enzyme from *P. sapidus* was expressed under induced conditions using Tween® 80, as the gene could be amplified from the cDNA or the protein was found by MS (Fig. S2). It was already demonstrated by several groups that the secretion of fungal and in particular basidiomyceteous enzymes is highly dependent on the culture conditions as the organisms have to adapt to the challenging environmental conditions (Filiatrault-Chastel et al. 2021; Gressler et al. 2021). However, normally large amounts of different enzymes are induced (Xie et al. 2016) and some of them have catalytic promiscuity to a certain degree (Leveson-Gower et al. 2019), so that the function and the mechanism of those enzyme are often predicted but not totally solved. Moreover, basidiomyceteous enzymes often do not have simple counterparts in the better studied Ascomycota (Liers et al. 2011; Brack et al. 2022). Therefore, the information on basidiomyceteous enzymes in databases is still underrepresented, making the predictions even more tedious. In regard of this, a function mining of the PSA_Lip was done, using database information about GDS(L) hydrolases, which were discovered since 1995 (Upton and Buckley 1995) and a vast amount of already known functions exists (Akoh et al. 2004). While using different kinds of predictions, only moderate identities to previously characterized GDSL enzymes phospholipase, acetylcholinesterase and acetylxylan esterase, from the carbohydrate esterase family 16, with 23.8–25.1%, 26.6% and 28.4% respectively, were found, giving no clear information about the function of the enzyme. In the literature, no distinct threshold for a precise identification is denoted. In general > 50% identity (Espadaler et al. 2008) is suggested, however sometimes lower identities may be sufficient, as e.g. shown for imine reductases with 29% identity (Gand et al. 2014). In contrast, even a high identity did not provide a clear distinction of function, as shown for two enzymes, a fatty acid isomerase and a fatty acid hydratase, which share 99.5% identity (Fibinger et al. 2016). If only the total sequences are compared, the resulting phylogenetic tree looks irregular for the known CE16 proteins, as they did not cluster into one particular clade (Fig. 2), but differences are known for bacterial and eukaryotic enzymes in the same CAZyme families (Chettri et al. 2020), especially for the CE16 family (Urbániková 2021). However, the great differences of the PSA_Lip-like proteins from the described enzymes is clearly obvious. To depict the differences even more, a motif analysis with (plant) GDSL hydrolases and a selection of CE16 GDS(L) type hydrolases was done. According to Akoh et al. (plant) GDSL hydrolases have five distinct sequence motif blocks (Akoh et al. 2004). Those motifs were investigated showing huge differences from the PSA_Lip-like enzymes from Basidiomycota (Fig. 3E) to known plant GDSL hydrolases (Fig. 3C), while higher similarities were found for CE16 GDS(L) type hydrolases (Fig. 3D). These findings fit very well to the in silico analysis of Urbániková, who identified five conserved blocks as well, while that work was focussed on all acetylesterases annotated in the CAZy database (Urbániková 2021). It was clearly shown that fungal CE16 have not a GDSL but a GDS(Y/W/F) motif, while GDSY is the dominating form of the motif (Fig. 3E, block 1). In block 3 the “conserved” GxND (position 6–9 in Fig. 3C, E, Block 3) is different from the PSA_Lip-like enzymes where asparagine is predominantly exchanged to glycine. In block 5, the already described enzymes exhibit a D/NxxH motif, including the deposited basidiomyceteous CE16 enzymes in the CAZY database. However, the PSA_Lip-like enzymes are clearly different here, having neither a histidine nor an aspartate or an asparagine. If the 3D model of the PSA_Lip is compared with the moderately similar enzymes, no other acidic or alkaline or polar residues except for D63, as part of the GDS(L) motif, (position 3 in the block 1 in Fig. 3C–E) are in a distance of < 5 Å. As no crystal structure of a CE16 enzyme is deposited in the PDB database, the PSA_Lip model is a computational prediction and the relevant motifs should, therefore, only be considered as a structural aid for the observed results. Interestingly, the extracellular GDSL lipase from *Streptomyces rimosus* (PDB: 4HYQ) has one short helix compared to the PSA_Lip (Fig. 4D, red circle), which allows N82 pointing to the catalytically active S10 in this enzyme. This residue is involved in oxyanion hole formation, while this enzyme still has the histidine in suitable distance to form a catalytic dyad (Leščić Ašler et al. 2017). A potential option for the PSA_Lip is that a “close” residue in the PSA_Lip can take over the function of the catalytic active histidine. Therefore, the distances of the S64 were analyzed (data not shown), but only D169 and D171 with 12 or 9.7 Å were identified. Those residues may work as a bridge to fix a water molecule, which hydrolyzes the enzyme–substrate complex, as they are in a relevant distance, especially if taken into account that the helix in which they are located could be shortened as in the *S. rimosus* lipase. Moreover, an induced fit mechanism for the GDSL lipases is known, which makes a precise analysis of potential residues in a homology model complicated, but at the same time underlines the potential involvement of nearby amino acid residues such as the mentioned D169 and D171. Therefore, potentially relevant residues should be investigated in more detail via structural determination and molecular dynamics analysis.

The expression of the PSA_Lip using different constructs yielded high activity against pNPO using the strain RH32919, where the *cbhI* promoter and *cbhI* terminator of *T.* *reesei* were used. An easy one-step SEC purification yielded a high purification factor of 36, and LC-ESI-HR-MS/MS confirmed the identity of the enzyme. In the industry, *T.* *reesei* is used as one of the working horses of the commercial production of different enzymes because of its generally high yields (Jørgensen et al. 2014).

The temperature and pH-optima of PSA_Lip were similar to those of enzymes with acetyl xylan esterase activity from *Schizophyllum commune* (Halgasová et al. 1994), *Streptomyces lividans* (Dupont et al. 1996) and *Bacillus pumilus* (Degrassi et al. 1998). Interestingly, it was not possible to fit a hyperbolic curve to the data for pNPP according to the Michaelis–Menten equation, so no kinetic data could be calculated, which raised the question, whether the natural functionality of PSA_Lip actually is typical lipase activity or an acetylesterase of acetylated carbohydrates (either from short oligomers or from polymeric carbohydrates such as xylan). One potential reason could be that the used substrate concentrations for pNPP were insufficient to reach the saturation state, however, higher substrate concentrations could not be measured in the used assay due to solubility issues. Beside hyperbolic fits, linear and sigmoidal fits were tested, resulting in an unreasonable K_M_ value of 25 ± 197 mmol L^−1^ and low determination coefficients (R^2^) of < 0.92. Additionally, the activity for the hydrolysis of a real lipid (trioctanoate) compared to the corresponding *para*-nitrophenol ester analog (pNPO) with a good leaving group was roughly 250 times lower, providing another indication that the actual substrates may not be triglycerides. As no activity of the PSA_Lip was found for the acetylated monosaccharides, no carbohydrate deacetylase activity could be claimed, but not completely excluded. Until now, at least two types of enzymes able to deacetylate the polymer xylan have been described: (I) acetyl esterases (AE; E.C. 3.1.1.6), which remove acetyl groups from short xylooligosaccharides (XOS) (Poutanen et al. 1990; Biely et al. 2011) and (II) acetyl xylan esterases (AXE; E.C. 3.1.1.72), which are able to deacetylate polymeric xylan and in addition XOS (Biely 1985; Poutanen et al. 1990). The data generated indicate that more research in that field has to be done to better understand the enzymes involved in the degradation of lignocellulosic biomass by Basidiomycota.

The information presented in this study is useful for identifying potential functions of the PSA_Lip and similar basidiomycetous enzymes due to their unusual SGGI motif. Their exact mechanism has to be further characterized with the help of structural determination and molecular dynamics and mutagenesis of different amino acid residues in the near future. Maybe, these enzymes will form a new CE family and will be applied at slight alkaline conditions due to their high stability at this pH range.

## Supplementary Information

Additional file1

## Data Availability

We conducted experiments and data were generated. All data is shown in Figures and Tables within the article.

## Abbreviations

CAZymes: Carbohydrate active enzymes
CE: Carbohydrate esterases
d: Days
DTT: Dithiothreitol
EMBOSS: European Molecular Biology Open Software Suite
ExPASy: Expert protein analysis system
HPLC–DAD: High performance liquid chromatography diode-array detection
LB: Lysogeny broth
min: Minutes
MALDI-MS/MS: Matrix-assisted laser desorption/ionization mass spectrometry
MOPS: 3-(*N*-Morpholino)propanesulfonic acid
ML: Maximum likelihood
MWCO: Molecular weight cut-off
PDB: Protein data bank
pNPA: *p*-Nitrophenyl acetate
pNPB: *p*-Nitrophenyl butyrate
pNPO: *p*-Nitrophenyl octanoate
pNPP: *p*-Nitrophenyl palmitate
PSA_Lip: *Pleurotus sapidus* lipase
PSI-BLAST: Position-specific iterative basic local alignment search tool
rpm: Rounds per minute
SDS-PAGE: Sodium dodecyl sulfate–polyacrylamide gel electrophoresis
SNS: Standard nutrient solution
TLC: Thin layer chromatography
T_50_^60^ value: Temperature at which enzymatic activity is reduced to 50% after 60 min
U: Units (µmol min^−^^1^)
v/v: Volume per volume
XOS: Xylooligosaccharides
