# Supplementary material for: Characterization of a GDS(L)-like hydrolase from Pleurotus sapidus with an unusual SGNH motif
Source: AMB Express. 2024 Sep 3;14:98. doi: 10.1186/s13568-024-01752-x (PMC11372007; doi:10.1186/s13568-024-01752-x)
Supplement: Supplementary file 1 — Additional file1 [file 13568_2024_1752_MOESM1_ESM.docx]

**Characterization of a GDS(L)-like hydrolase from *Pleurotus sapidus* with an unusual SGNH motif**

Miriam A. Fingerhut^1^, Lea Henrich^1,^ Christiane Lauber^2^, Niklas Broel^1^, Parviz Ghezellou^2^, Dominik Karrer^1^, Bernhard Spengler^3^, Kim Langfelder^4^, Timo Stressler^4^, Holger Zorn^1,5^, Martin Gand^1,^*

^1^Institute of Food Chemistry and Food Biotechnology, Justus Liebig University Giessen, Heinrich-Buff-Ring 17, 35392 Giessen, Germany

^2^Hochschule Fresenius - University of Applied Sciences, 65510 Idstein, Germany

^3^Institute of Inorganic and Analytical Chemistry, Justus Liebig University Giessen, Heinrich-Buff-Ring 17, 35392 Giessen, Germany

^4^AB Enzymes GmbH, Feldbergstrasse 78, 64293 Darmstadt, Germany

^5^Fraunhofer Institute for Molecular Biology and Applied Ecology, Ohlebergsweg 12, 35392 Giessen, Germany

*Corresponding author

E-mail: martin.gand@lcb.chemie.uni-giessen.de; ORC-ID: 0000-0001-8211-691X

**The codon optimized sequence of the PSA_lip**

ATGCTCCGCTCCTTCGTCGTTCTGACTTCCGTGTGCGCTGCCTACGCGGCGGTTCTGCCCCGCGACGCACCCAACGGAGTTCACCTTGCGGTCGACCCTAAGTGCGGTGTGGCAGGTGGCAGGTTTGGCGATGTCAACATCGGACTGAAGCCGCTCACGAGCTACGAGCACATCGTCGCGTTTGGCGATTCGTGGACTGACGGAGGAGCTCACAACGGAGAACCACTGCCACCCCCTGTACTGACCCCCCCTAATCCGAGGGCTGGTGGACGGGCTTCAAACGGTCCCGTTTGGGTCGAGAAGCTTGCTTCTGCAGCTGGGGCTACCCTTCTCGACTTCGCTGAGATTGGCGCAGTCACCGATAAGAACATCTGGCCCAGCTCGCTGCTTCCTACTACCGCTTCGTCCGCCAACGACTTTGTCGGTCAGGCCCACAACTACATTAACCAGCGAAACGGCTTTGATCCGGAGACGACCCTCTACACCATTTTCCTGGGAGTCGGCGACTTCGACCTGAGCCAACAGACTGGCACGGATAACCTCTACACGGTGGCTGGCGCCATTGTCTATACGATCCTGGAGCTCACCAGCTACCCCACCTACGCCAAGAACATTATCGTCGTTGACAACTACGGTCGTGGGATCTACGAAACCCCGTCC

GGAGACGCGTTCAAGGAGGGGATCTACGCTGGCCTCAACACACTCCACACACGCTACGGCACTTCCGTCGGATTCGTCGACCTCAAGACCCTTTGGGACGGCGTCCTCGGAAGCTCACCAGGATACGAGGCCTTTGGCTACACCTCTAAGGCGGCCTGCTTGCCTTCTAGCACGTCGACGTCTGGAGCCTGTGCCAACCCTGAGTCCACGTTCTACTGGCTCCCCGGCATCCCGTCGGCAGCTACTCACGGTCTGATCGCGGACTACGTGGAGAAGGTCCTGACGACCTGCTGA


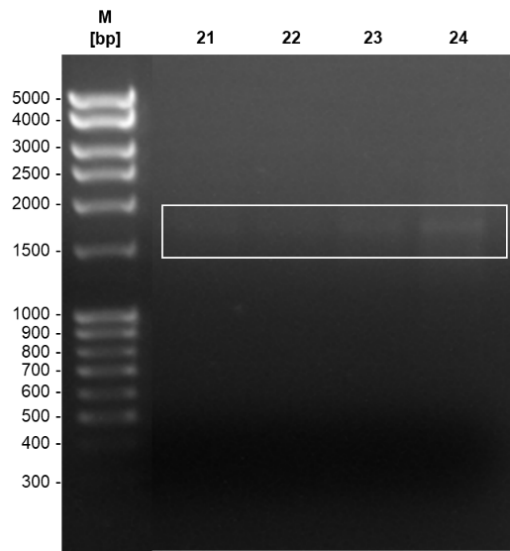


**Fig. S1.** 1.2% agarose gel of *P*. *sapidus* cDNA libraries after 21-24 cycles. M = 100 bp extended by Roth.


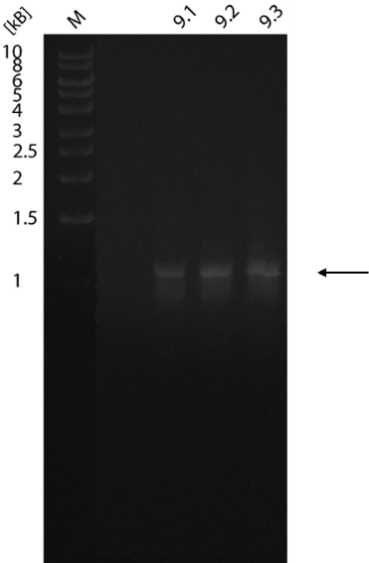


**Fig. S2.** 1.2% agarose gel of PSA_Lip DNA amplified from *P*. *sapidus* cDNA libraries. M = 1 kb ladder by Roth.


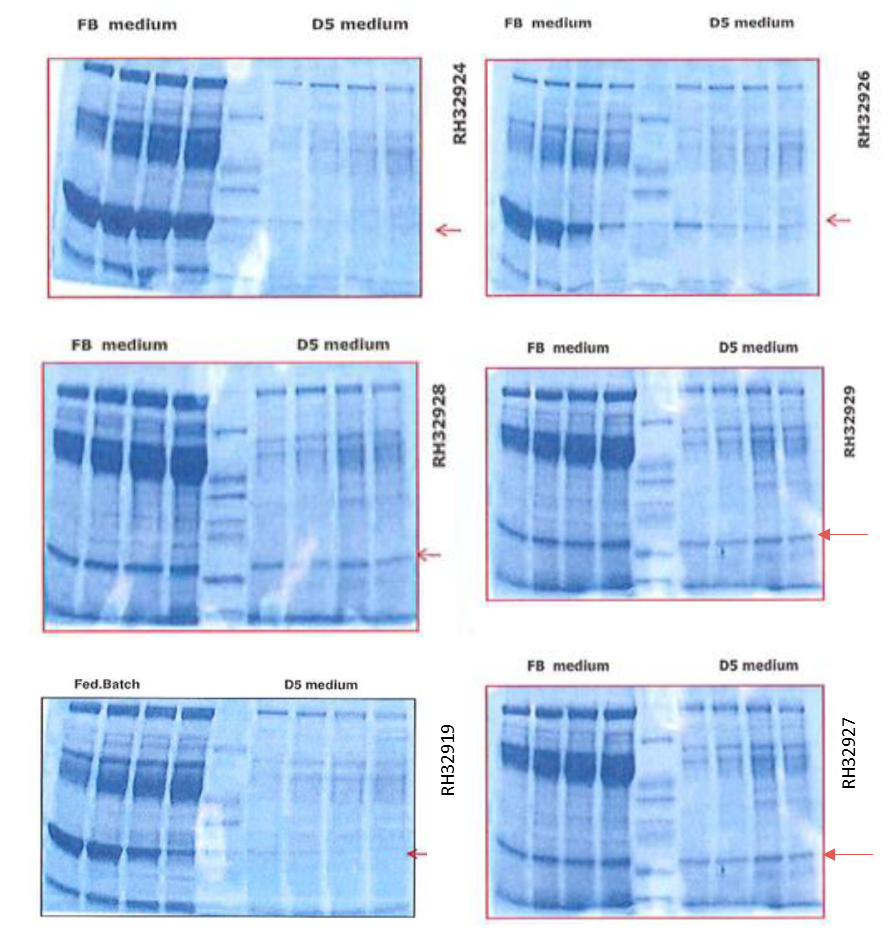


**Fig. S3.** Denatured SDS PAGE pictures of the expression of the PSA_Lip from the different *Trichoderma reesei* strains. The red arrow marks the theoretical MW of the PSA.


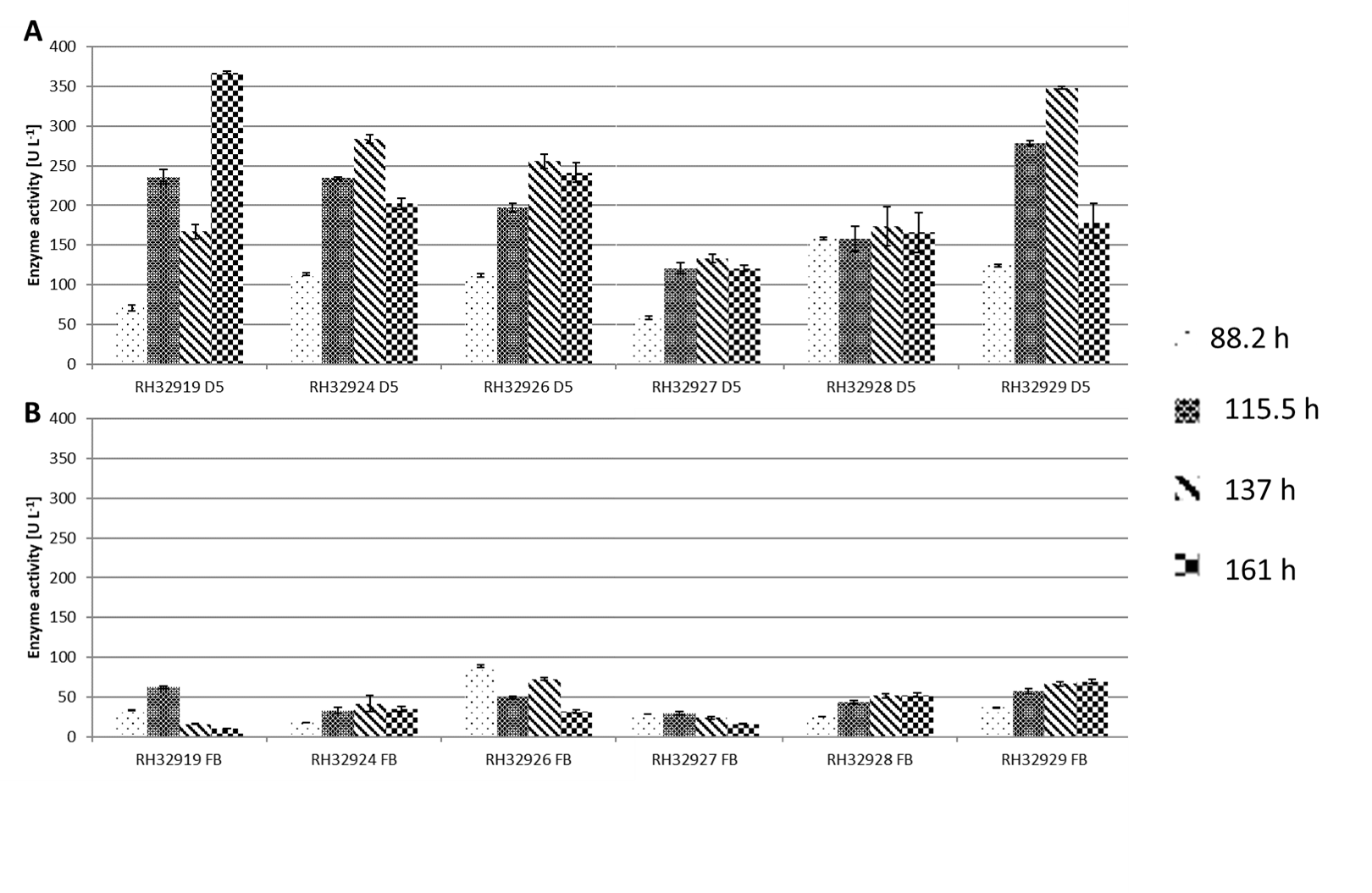


**Fig. S4.** pNPP activities of the PSA_Lip produced recombinantly in strains RH32919, RH32924, RH32926, RH32927, RH32928, RH32929 in the medium D5 (**A**) or in fed-batch (**B**).


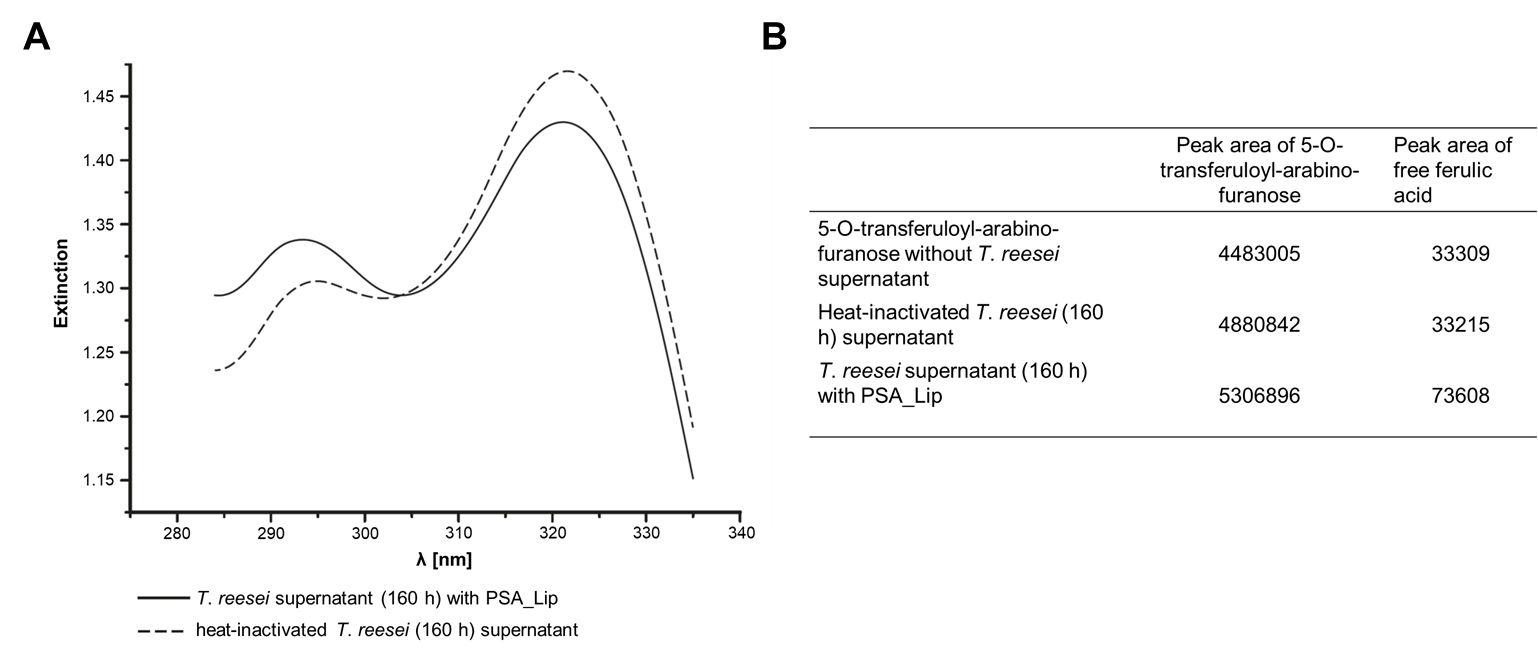


**Fig. S5.** Feruloyl esterase activity of PSA_Lip: UV/Vis spectrum (280-340 nm) after 72 h of incubating PSA_Lip with ferulic acid methyl ester (**A**) and HPLC-DAD peak areas of 5-*O*-transferuloyl-arabino-furanose and free ferulic acid (**B**).


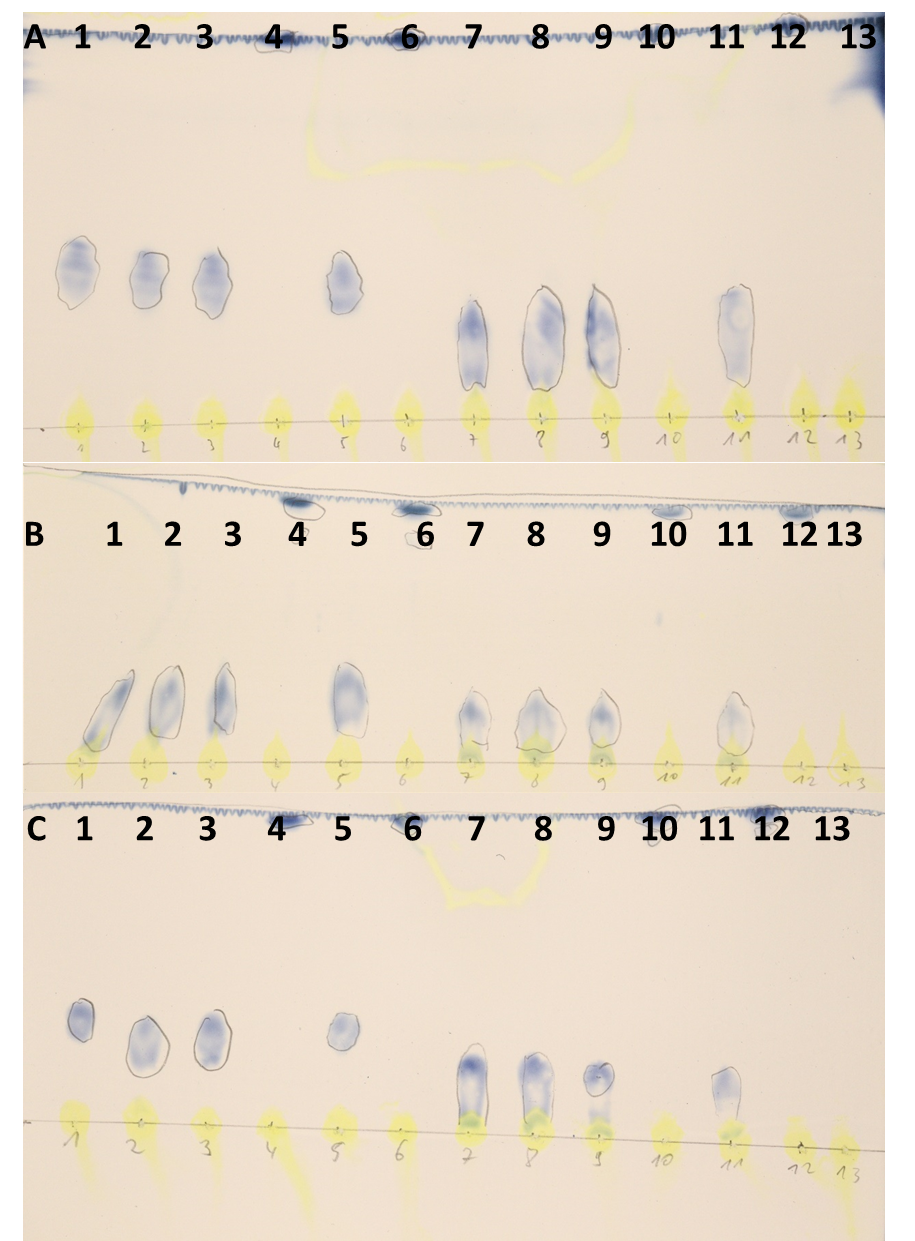


**Fig. S6.** Thin layer chromatography analysis of the conversion of d-xylofuranose tetraacetate and α‑d‑(+)-glucose pentaacetate by the PSA_Lip and a commercial acetylxylan esterase from *Orpinomyces* sp. from different time points. (**A**) 15 min, (**B**) 4 h and (**C**) 24 h, lanes 1 = Xylose + buffer, lane 2 = Xylose + acetylxylan esterase, lane 3 = Xylose + PSA_Lip, lanes 4 = d-Xylofuranose tetraacetate + buffer, lane 5 = d-Xylofuranose tetraacetate + acetylxylan esterase, lane 6 = d‑Xylofuranose tetraacetate + PSA_Lip, lanes 7 = Glucose + buffer, lane 8 = Glucose + acetylxylan esterase, lane 9 = Glucose + PSA_Lip, lanes 10 = α‑d‑(+)-Glucose pentaacetate + buffer, lane 11 = α‑d‑(+)-Glucose pentaacetate + acetylxylan esterase, lane 12 = α‑d‑(+)-Glucose pentaacetate + PSA_Lip and line 13 only buffer.


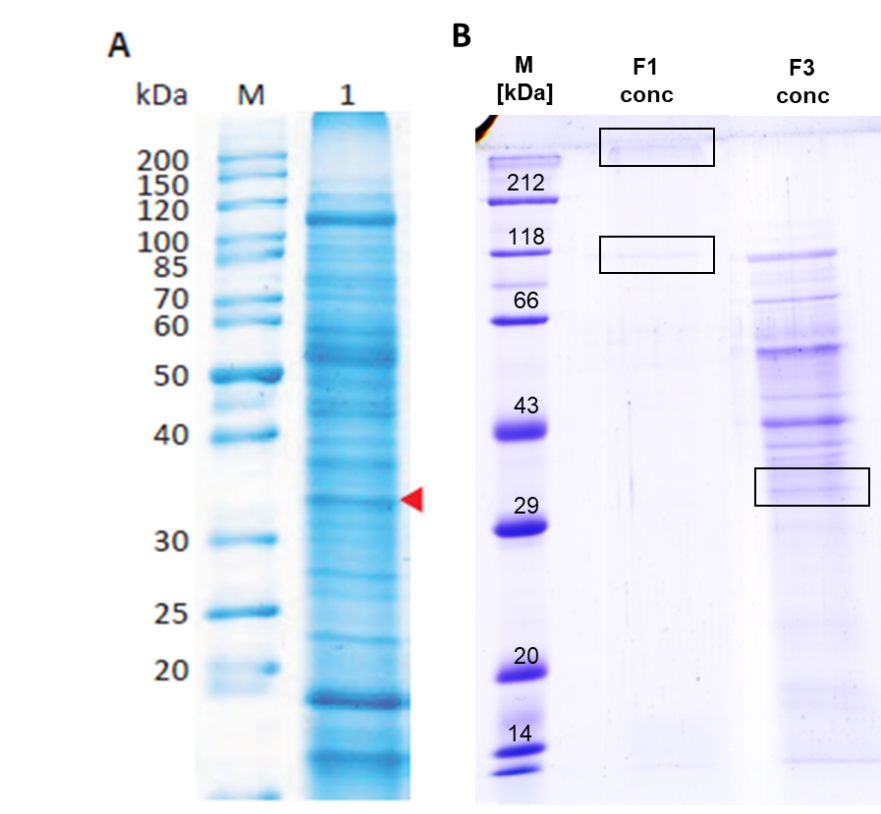


**Fig. S7.** Electrophoretic analysis of the culture supernatant with expressed PSA_lip; (**A**) Denatured 12% SDS-PAGE, M - Protein standard Carl Roth, red arrow marks PSA. (**B**) Semi-native SDS-PAGE of the SEC purification M – Protein standard – Thermo Fisher Scientific.


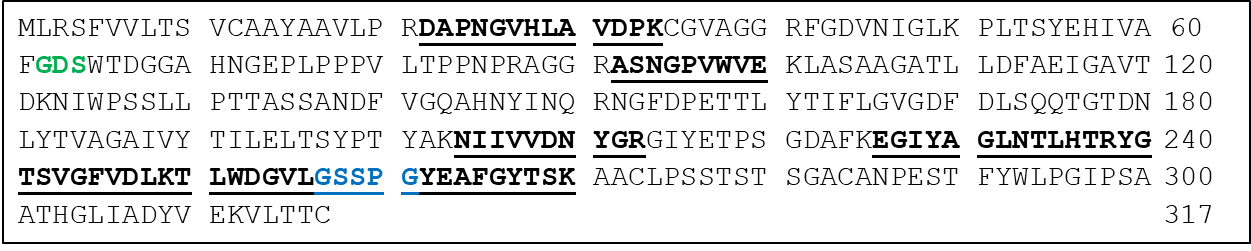


**Fig. S8.** Sequence of PSA_Lip. Bold, underlined amino acids indicates peptide fragments identified by LC-ESI-HR-MS/MS after gel digestion of purified PSA_Lip fraction F1_1. Potential active site sequences are highlighted in green (GDS(L) motive) and blue (GxSxG motive).


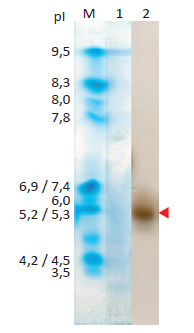


**Fig. S9.** Isoelectric focusing for the determination of the pI of the recombinant PSA_Lip. M - Protein standard Carl Roth, 1 - Colloidal Coomassie staining, 2 - Activity staining with α-naphthyl acetate.

**Fig. S10.** Activity measurements of PSA_Lip toward pNPP.

**
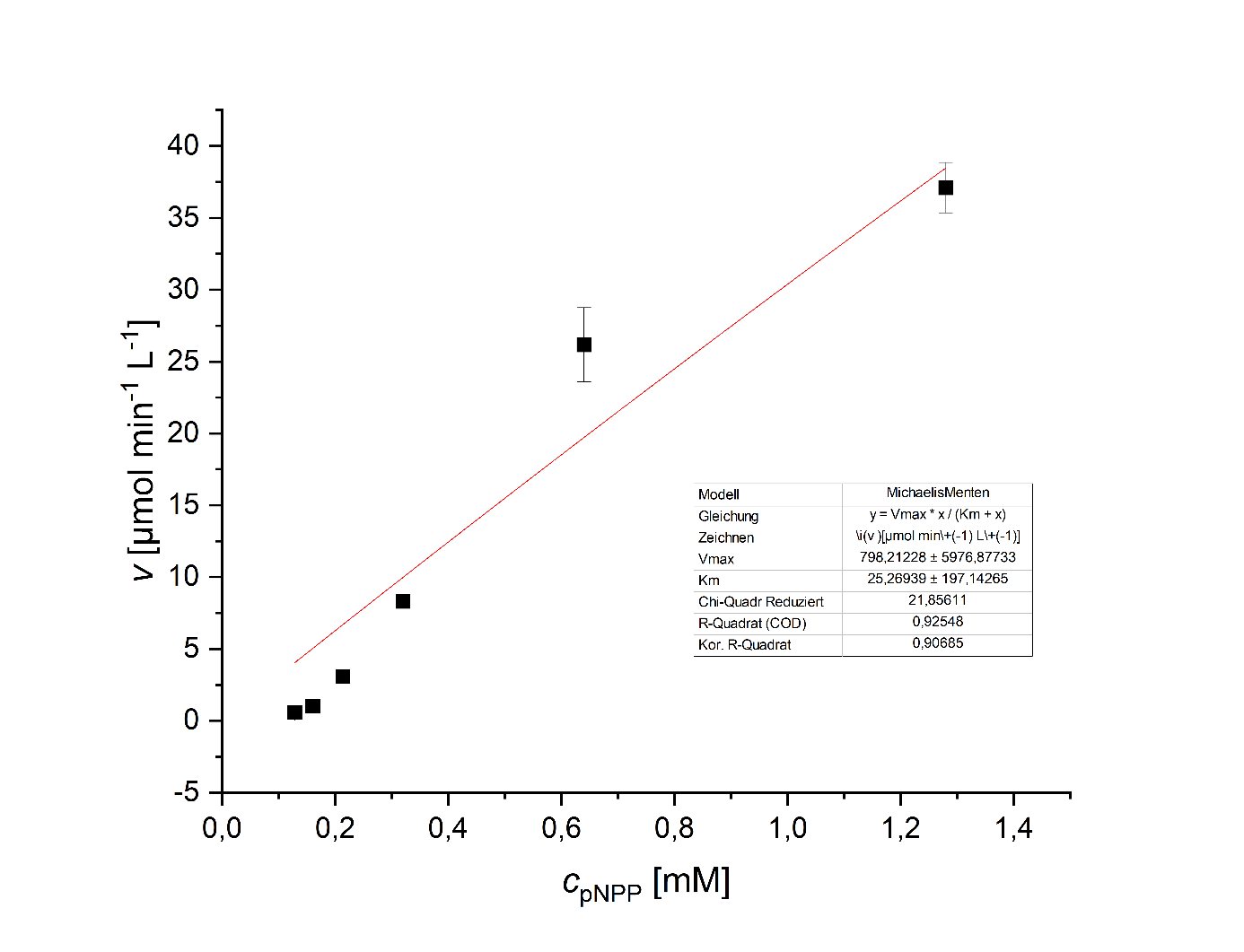
**

**Table S1.** Primer sequences for amplifying the PSA_Lip

| **Primer name** | **Sequence** | **Melting temperature [°C]** | |
| --- | --- | --- | --- |
| GDSL-like Lipase for1  GDSL-like Lipase rev1 | 5'- ATGCTCCGATCCTTTGTCG -3'  5'- TCAGCATGTTGTCAGCACC -3' | | 58  58 |

**Table S2.** Activity measured for the PSA_Lip towards trioctanoate

| **Time** | **Specific activity [U mg^-1^]** |
| --- | --- |
| 1 h  2 h  4 h | 11.2 ± 1.6  8.8 ± 0.3  8.3 ± 0.3 |
